# Muscle Activation during the Squat Performed in Different Ranges of Motion by Women

**DOI:** 10.3390/muscles2010002

**Published:** 2023-01-12

**Authors:** Lissiane Almeida Cabral, Leonardo Coelho Rabello Lima, Christian Emmanuel Torres Cabido, Rogério César Fermino, Saulo Fernandes Melo Oliveira, Alexandre Igor Araripe Medeiros, Luis Fabiano Barbosa, Thiago Mattos Frota de Souza, Túlio Banja, Cláudio de Oliveira Assumpção

**Affiliations:** 1 Post-Graduation Program in Physiotherapy and Functioning, Federal University of Ceará, Fortaleza 60430-235, CE, Brazil; 2 School of Physical Education and Sport of Ribeirão Preto, University of São Paulo, Ribeirão Preto 14040-907, SP, Brazil; 3 Post-Graduation Program in Physical Education, Department of Physical Education, Federal University of Maranhão (UFMA), São Luís 65080-805, MA, Brazil; 4 Research Group in Environment, Physical Activity, and Health, Physical Education Graduate Program, Federal Technological University of Paraná, Curitiba 80230-901, PR, Brazil; 5 Physical Education Graduate Program, Federal University of Paraná, Curitiba 81530-900, PR, Brazil; 6 Physical Education Graduate Program, Vitória Academic Center, Federal University of Pernambuco, Recife 50800-220, PE, Brazil; 7 Research Group in Biodynamic Human Movement, Institute of Physical Education and Sports, Federal University of Ceará, Fortaleza 60455-760, CE, Brazil; 8 Independent Researcher, Passos 37900-577, MG, Brazil; 9 Applied Kinesiology Laboratory–LCA, School of Physical Education, University of Campinas, Campinas 13083-959, SP, Brazil; 10 Department of Sport Sciences, Institute of Health Sciences, Federal University of Triângulo Mineiro, Uberaba 38025-350, MG, Brazil

**Keywords:** electromyography, rectus femoris, biceps femoris, gluteus maximus, vastus lateralis

## Abstract

Purpose: To analyze the muscle activation of the rectus femoris (RF), vastus lateralis (VL), gluteus maximus (GM), and biceps femoris (BF) in concentric and eccentric actions in the squat at 90° and 140° range of motion. Methods: Thirty-five women (32.9 ± 7.4 years; 64.5 ± 11.5 kg; 1.63 ± 0.1 m; BMI: 24.2 ± 2.9 kg/m^2^; %fat: 24.9 ± 6.5%) experienced exercise for at least eight weeks. Electrodes were positioned in standardized locations. The signals were acquired by an A/D SAS1000 V8 converter and the electromyographic activity normalized in the percentage of the highest produced value (%RMS). The data were analyzed using repeated measures two-way ANOVA, with effect size (η^2^) and differences calculated in percentage points (∆ p.p.). Results: The RF (*p* = 0.001; ∆ = 5.1 p.p.) and BF activation (*p* = 0.020; ∆ = 4.0 p.p.) was higher at 90° in the eccentric action. The RF showed an interaction between the range of motion and %RMS, with a large effect size (F = 37.9; *p* = 0.001; η^2^ = 0.485). The VL activation was higher at 140° (*p* = 0.005; ∆ = 3.9 p.p.) in the concentric action and higher at 90° (*p* = 0.006; ∆ = 3.7 p.p.) in the eccentric action, with a large effect size significant interaction (F = 21.3; *p* = 0.001; η^2^ = 0.485). The GM activation was higher at 90° in the concentric (*p* = 0.020; ∆ = 5.4 p.p.) and eccentric action (*p* = 0.022; ∆ = 41 p.p.). Conclusions: The biarticular muscles were influenced by the squat range only in the eccentric action of the movement, while the monoarticular muscles were influenced by the squat in both concentric and eccentric muscle action.

## 1. Introduction

The squat movement recruits distinct muscle groups that involve the hip, knee, and ankle joints, consisting of one of the most common exercises in strength training (ST) programs [1]. In addition, this exercise is used as a form of rehabilitation because it enables the strengthening of the hip and thigh muscles [2] and presents a movement with a mechanical–functional pattern [3]. In this sense, the understanding of muscle activation may assist in improving training prescription and, consequently, the generated adaptations, contributing to physical performance, quality improvement, and performance of activities of daily living [1]. Adjustments are necessary in training variables regarding the improvement in physical performance, such as the type and order of exercises, execution speed, types of muscle contraction, and range of motion [4].

Although the squat has been identified as one of the most used exercises for the development of lower limb strength and power, including in sports [5,6,7,8], studies have shown that this exercise may not be ideal for reducing imbalances between the muscle groups of the hamstrings and quadriceps, regardless of their range, as different ranges of the squat movement do not generate higher activation of the posterior muscles of the thigh [2,9]. However, Sousa et al. [10] demonstrated that a higher range of the squat causes a higher divergence of force between the rectus femoris (RF) and biceps femoris (BF) muscles, as the activation of the anterior thigh muscle is higher with increasing range. These findings agree with those found by Gorsuch et al. [9] when demonstrating increased activation of the RF muscle when comparing the squat at 45° with 90°, requiring performing specific exercises for the posterior muscles of the thigh, such as leg curl machine and stiff.

Experiments conducted exclusively with male volunteers showed variations in the range of motion lead to changes in strength development, activation, and synchronization of motor units [11,12]. However, men and women have different strategies to perform the squat in addition to the range of motion, with women presenting greater knee flexion and men presenting greater trunk flexion while performing the squat with greater range of motion [13]. Still regarding squat kinematics, differences between men and women were reported in the hip joint amplitude (greater flexion) and knee joint amplitude (increase in valgus). The biomechanical differences between males and females during the squat may result from differences in lower extremity ranges of motion, which really suggest that there are different strategies between the sexes during the execution of the squat [14]. Women show an emphasis on the knee joint, which produces more torque and, consequently, higher activation of the quadriceps muscle group [15]. Specifically in the BF muscle, a difference was found in the electromyographic activity of this muscle in the comparison between the sexes, being lower for women [16]. Thus, women present higher activation of the anterior muscle of the thigh during the squat, and this exercise is not recommended when the objective is to strengthen the posterior muscle of the thigh [17]. The information related to women does not fully contemplate the analyses in different ranges of motion, which still needs to be investigated, as it has already been shown that this can result in different activations in men [18].

The relationship between the manifestation of strength and anterior and posterior thigh muscle groups is well established, being higher in the quadriceps muscle group. However, there are gaps to be better clarified in the relationships between the range of motion, activation, and muscle action. Contreras et al. [19] found no differences in the activation of the vastus lateralis (VL), gluteus maximus (GM), and BF muscles relative to the range of motion in the squat. To our knowledge, this is one of the few studies that try to relate these variables exclusively to females, but the studies did not report in their findings the relationship between activation and muscle actions [13,18,20].

Knowledge about neuromuscular changes in terms of muscle activation due to the range of motion of the squat is essential and scarce, especially in women [19]. Since there is evidence indicating differences in muscle activation between young men and women, even when performing the same exercises [14,16], extrapolation of studies results conducted exclusively on men to women is inappropriate. This study aimed to investigate whether there is a difference in the activation of the RF, VL, GM, and BF muscles relative to the squat range (90 vs. 140° of knee flexion) in young women. These muscles were chosen due to their unique characteristics, with RF and BF being biarticular antagonistic muscles acting in the hip and knee joints, while the GM is a monoarticular muscle acting in the hip joint and the VL is also monoarticular but acts exclusively in the knee joint. The tested hypothesis was that different squat ranges would result in different activations in the studied muscles, especially the single-joint ones.

## 2. Results

Participants had a mean of 8.0 (±1.0 h) and 7.0 (±1.0 h) hours of sleep on the nights before strength tests on the first and second days, respectively. The subjective perception of recovery (SPR) measured before the 10 maximal repetitions (MR) test was 9.0 (±2.0) and 8.0 (±1.0) points for the first and second day, respectively. The mean load was 68.0 (±15.5 kg) and 60.1 (±17.9 kg) for the movement performed at 90° and 140°, respectively (*p* < 0.05).

**Figure 1:**
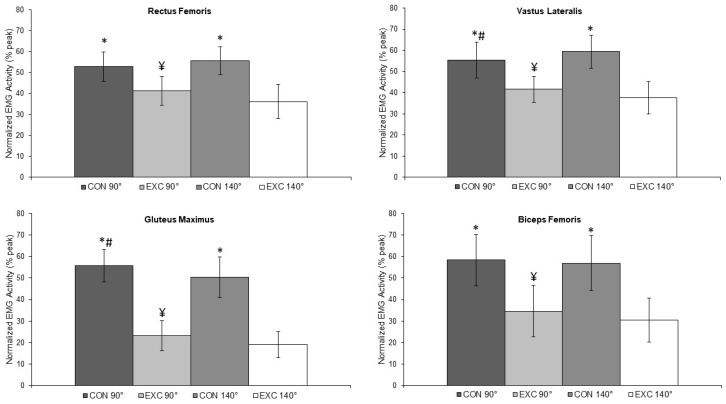
Muscle activation during the squat performed by women at 90° and 140° of range of motion * Differences between concentric and eccentric actions; ¥ Differences between eccentric actions; # Differences between concentric actions.

Table 1 shows the %RMS values for each muscle action (concentric and eccentric), range of motion (90° and 140°), and the difference between ranges of motion in percentage points (∆). Figure 1 shows the %RMS values.

RF showed no significant difference in %RMS in the concentric action at 90° or 140° (*p* = 0.097; ∆ = 2.8 p.p.). Muscle activation in the eccentric action was higher at 90° (*p* = 0.001; ∆ = 5.1 p.p.). An interaction was observed between the range of motion and %RMS, with a large effect size (η^2^ = 0.527). VL showed a higher %RMS at 140° (*p* = 0.005; ∆ = 3.9 p.p.) in the concentric phase. However, activation in the eccentric action was higher at 90° (*p* = 0.006; ∆ = 3.7 p.p.). An interaction was observed between the range of motion and %RMS, with a large effect size (η^2^ = 0.485).

GM showed a higher %RMS at 90° both in the concentric (*p* = 0.020; ∆ = 5.4 p.p.) and in the eccentric action (*p* = 0.022; ∆ = 4.1 p.p.). However, activation in the eccentric action was higher at 90° (*p* = 0.006; ∆ = 3.7 p.p.). No interaction was observed between the range of motion and %RMS (*p* = 0.409), with a small effect size (η^2^ = 0.020). Finally, BF activation was similar in the concentric action (*p* = 0.055; ∆ = 1.5 p.p.). The eccentric action showed a higher %RMS at 90° (*p* = 0.020; ∆ = 4.0 p.p.). No significant interaction was observed between the range of motion and %RMS (*p* = 0.902), but the effect size was medium (η^2^ = 0.026).

## 3. Discussion

This study aimed to investigate whether there is a difference in the activation of the RF, VL, GM, and BF muscles in terms of squat range in young women. The results confirmed our hypothesis and showed that only the evaluated monoarticular muscles were influenced by the squat range and type of action. The VL and GM muscles act on the knee and hip joints, respectively, during the squat. However, these muscles are independently activated in only one joint at a time despite being synergistic throughout the movement.

VL showed higher activity during 140° squats in the concentric action. It agrees with a study carried out by Jaberzadeh et al. [21], who found higher VL activation with increasing squat range. The higher VL activation during the concentric action of 140° squats occurred because women perform higher knee flexion when the objective is to perform greater squat depth than men [13]. According to these authors, women tend to flex the knee more at the expense of the hip joint when performing 140° squats. In this sense, the smaller knee angle during 140° squats would cause a higher demand on the quadriceps muscle group [22] so that the higher squat depth would cause greater effort of these muscles, even with low loads, of approximately 50% 1 MR [23].

Our results differed from those in the study of Contreras et al. [19] who found no differences between the two ranges of the squat for the VL muscle. These differences may have occurred due to these authors [19] not having controlled the execution speed, despite adjusting the load for each type of squat, in their study. This may have influenced mainly the time under muscle tension between squats. Another point to be considered is that in their study, concentric and eccentric muscle actions were not analyzed separately, thus concentric actions may have overlapped eccentric actions, influencing the result. We can speculate that greater control of execution speed and definition of muscle actions would be more sensitive to identify differences in EMG activity.

VL was more activated at 90° in the eccentric muscle action. It occurred because this muscle has a higher moment arm during this action [24], which occurs when the knee angle is around 90° [19]. Moreover, the higher activation during the eccentric action could be justified by the higher magnitude of the used load since smaller ranges allow the use of higher loads [25] and, consequently, lead to higher muscle activation [20].

The change in range for GM generated higher muscle activation at 90° in both actions (i.e., concentric and eccentric). Da Silva et al. [23] found similar results, demonstrating higher GM activation at 90°. According to these authors, the higher activation at 90° can be justified by the presence of a longer arm moment at the hip, creating a higher hip extensor moment during concentric action at 90°, while the contractile capacity of the muscles would be reduced at 140°, mainly the monoarticular muscles, so that GM has an optimal length–tension relationship at 90° [2,18]. In addition, the lower gluteal activation due to an increase in the squat range may occur because GM is not necessary to maintain stability or allow higher hip flexion [26].

Our findings differ from those of Contreras et al. [19], who found no differences in the electromyographic activity of GM muscles when comparing squats. However, the movement phases in their study were not evaluated separately, a fact that may have influenced the EMG results. As already discussed, these authors used the same external load for both ranges. This might have led to changes in the time under tension and, consequently, GM activation [25], as stronger muscles could compensate for the activation of weaker muscles under submaximal load conditions, causing changes in EMG signals [18].

The higher activation of the GM muscle at 90° in our study can be explained by the higher magnitude of the used load. Load reduced when the squat depth increased, which was also reported by Flores et al. [25]. In turn, higher loads seem to influence higher kinetic stimulus and higher activation for this muscle [20]. This principle is supported by Morton et al. [27] and Looney et al. [28], who claim that higher loads provide greater recruitment of motor units, based on the higher range of the electromyographic signals compared to lighter loads. The same behavior was identified during eccentric actions.

Both muscle actions showed differences for the two types of squats, with higher activation in the concentric action. Gullet et al. [1] and Clark, Lambert, and Hunter [29] found similar results. According to Robertson, Wilson, and Pierre [26], GM is less activated at 140°, as the moment of force of extensor muscles during the descent eccentrically controls hip flexion while allowing this joint to perform flexion. It leads to a reduction in activity as the squat reaches its maximum depth.

The biarticular muscles investigated in our study (i.e., RF and BF) showed differences in muscle activation only in eccentric actions at 90°. These muscles have agonistic action at one joint and antagonistic action at the other [30]. There is an intermediate activation of these muscles when this occurs. It means that the muscles obey a pre-established coordinating pattern for the task. Thus, it seems that the range would not influence this pattern and, consequently, the activation of the RF and BF muscles. Another point to be considered is that these muscles would act as knee stabilizers, plus they need to be neutralized by other muscles to avoid unwanted actions during the movement [31]. Hip and knee extension during the squat is the result of the differential moment arms of the two muscles at each joint and range of motion [24].

Regarding the range of motion and relative muscle effort, a higher range of the squat resulted in a higher effort of the knee extensor muscles. Furthermore, the hip extensor muscles manifested higher effort as load progressed accompanied by the increased range of motion [23].

Our results showed similar RF activation during concentric action regardless of range. These findings confirm the studies by Marchetti et al. [2] and Trindade et al. [18], who identified no differences in RF activation at 90° and 140° knee angles in the isometric squat. It was also verified for the dynamic squat [24,32]. As reported, the increase in the squat range may not interfere with muscle activation due to the biarticular RF function. In this case, this muscle acts as a hip flexor and knee extensor, thus reducing its activation when the hip is flexed during the squat [33].

Da Silva et al. [24] demonstrated that RF has as its main function to provide knee stabilization during the squat. In addition, RF has agonist action in the knee joint and antagonistic action in the hip joint during the concentric action, showing passive insufficiency in the knee joint and active insufficiency in the hip joint during movement [18].

Regarding the eccentric action, our results demonstrate that RF was more activated at 90°. The justification for it could be that the RF muscle length–tension relationship is influenced by the trunk positioning, as a trunk under higher flexion would reduce RF length and, consequently, its activation [32]. Moreover, RF has a higher strength arm when crossing the knee joint due to the patella, and it allows a higher rotational component of the knee joint around 90° [2], minimizing the action on the hip joint also in the concentric action.

The BF muscle showed no difference in muscle activation relative to the squat range in the concentric action. Our results corroborate those of Contreras et al. [19], however, this did not occur for the VL and GM muscles comparing the studies. This may be in part because biarticular muscles are less susceptible to the method adopted. In fact, the absence of difference in BF activation relative to the squat range may have occurred because the electromyographic activity of the hamstring muscles does not accompany the changes in the angles of the hip and knee joints [34]. Moreover, the BF is responsible for stabilizing the knee joint, as well as having a longer moment arm at the hip, providing a hip extensor moment [2].

The BF muscle showed higher activation at 90° in the eccentric action. Our results corroborate the study by Yavuz et al. [35], as the eccentric actions obtained lower activations than the concentric actions for the squat. Coratella et al. [32] stated that a higher electromyographic activity during the concentric action compared to the eccentric action can be justified by the lower speed of conduction of the nerve impulse in the muscle fibers in the eccentric actions during the execution of the squat [36], in addition to the contribution of non-contractile elements to the production of passive force [37], also occurring in other variations of the exercise [38]. This decrease in activation in eccentric action compared to concentric action seems to follow the same understanding of GM muscle behavior. Its action should be optimized in the hip joint, allowing its flexion during the descending phase. Furthermore, this biarticular muscle would undergo the influence of its antagonist (RF), decreasing its activation in 140° squats, as previously discussed in the GM muscle.

The present study has some limitations that should be considered. Although all participants were familiar with the squat in both ranges, performing the movements at a certain cadence was not part of the training routine and it could influence the intramuscular coordination and, consequently, the EMG data. Another point concerns the time under tension for each type of execution. Although the cadence was the same, the tension time at 90° was different at 140°, which could affect a different perception of effort during the test performance. However, we believe that the gain in ecological validity resulting from the investigation of the different phases of movement during dynamic actions compensates for these limitations. Furthermore, considering the peak activation for each muscle group could allow further insights regarding the relationship between range of motion and activation. Finally, future studies should also collect morphometric parameters of the participants, which would allow for better conclusions regarding the differences observed between muscle activity in men and women considering not only sex.

## 4. Materials and Methods

This was a cross-sectional study, approved by the Institutional Committee for Ethics and Research in Human Beings, under protocol number 4.277.406. Written consent was obtained from all participants before any intervention.

### 4.1. Participants

The sample of the present study consisted of 35 women (age: 32.9 ± 7.4 years; body mass: 64.5 ± 11.5 kg; height: 1.63 ± 0.1 m; BMI: 24.2 ± 2.9 kg/m^2^; fat percentage: 24.9 ± 6.5%). The sample size was determined from the calculation performed in the software G*Power v 3.1.9.6 (G*Power, Kiel, Germany), using a mean difference of 5% and adjusting the power of the statistical test to 0.96. Participants who had an experience of at least two uninterrupted months with ST, back squat movement in their training plan, and passed the previous biomechanical assessment of the execution of the movement performed by the same evaluator were included in the study.

The following exclusion criteria were adopted: presenting any physical limitation or disability, answering “Yes” to any of the questions on the Physical Activity Readiness Questionnaire, presenting pain during squatting or knee and/or hip injuries diagnosed by orthopedic physicians, having undergone knee and/or hip surgery, having lesions in the spine region or postural deviations diagnosed by an orthopedic physician, and claiming to use anabolic steroids. However, there were no exclusions for any of these reasons.

An assessment of body composition was performed by measuring the thickness of skinfolds (SF) using a skinfold caliper (Sanny, São Paulo, Brazil) and a protocol of seven SF to characterize the sample [39]. Anamnesis was applied to identify the training period and possible limitations for the test application and the estimated load that each participant would start the test of 10 maximum repetitions (10 MR).

### 4.2. Experimental Protocol

**Figure 2:**
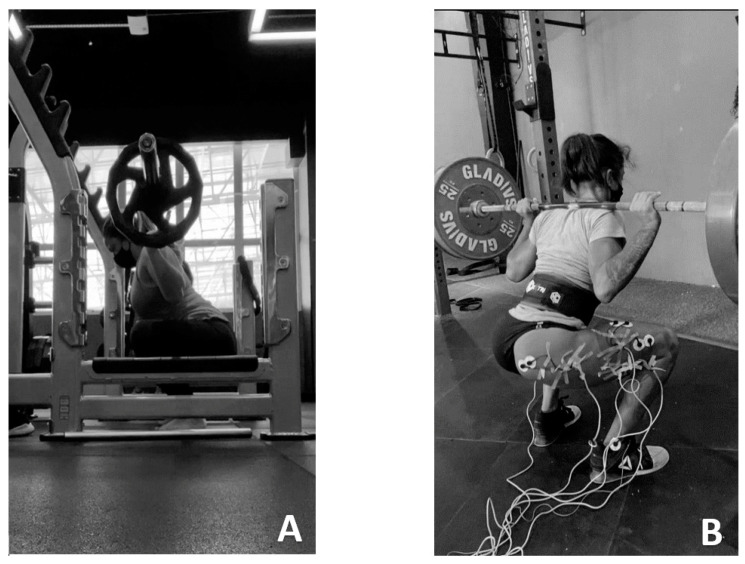
(**A**) Strength test (10 RM); (**B**) Electromyographic analysis during the squat.

The volunteers participated in three days of collection, with 48 h rest intervals. All of them were instructed not to exercise during the study period. The first and second days aimed to characterize the sample and determine the load for 10 maximum repetitions (10 MR), according to the joint range: 0–90° of knee flexion and 0–140° of flexion. On the third day, the experimental protocol was applied and the electromyographic signal was collected (Figure 2).

For the standardization of the intervention protocol, volunteers were given the following instructions: their feet should be positioned at hip width and vertically with the barbell, which should be positioned over the shoulders, which characterizes the back squat. The squatting movement should begin with the body in the orthostatic position and full extension of the knees and hips. A slight flexion of the trunk forward should be performed, which simultaneously reduces the joint amplitude of the knees, as well as of the ankles. Squats should be executed to the desired amplitude (0–90° or 0–140° of knee flexion), characterizing the eccentric phase, followed by return to the initial position, considered the concentric phase of the movement [33]. The preparatory phase was performed with the goniometer for the participants to become familiar with the amplitude of each squat, and in both exercises the volunteers were asked to perform the concentric and eccentric actions in two seconds (1:1), which were controlled by a metronome. There was no verbal command besides counting the number of repetitions.

A 10 MR test and retest were performed to determine the intensity that would be used for each squat range. In the first moment, the participants performed a preparatory phase consisting of 20 repetitions of air squats without weight for both ranges. After the preparatory phase, participants performed the 10 MR test for the deep squat. Each volunteer had up to three attempts to find the load for 10 consecutive squat repetitions. There would be a load increase of three to five percent if a higher number of repetitions were performed [40]. There was a five-minute rest between each attempt. The 10 MR test for 90° was performed after 30 min from the end of the 10 MR test for 140°. The applications happened in the same way, except for the preparatory phase. The volunteers performed the retest of the 10 MR load for both squats after a 48 h interval, following the same protocol of the first day from the preparatory phase to the end. However, the test load already started with the estimated load of the first day.

The experimental sessions were carried out in two stages on the third day, as previously mentioned, following a crossover design, with randomization performed by drawing lots to determine the initial squat condition. The specific preparatory activity was performed in the range of the selected squat by performing 10 repetitions with 50% of 10 MR, as established in the 10 MR test. The analysis of the electromyographic signals began after a two-minute interval during the performance of the squat at 90° and 140°, with an equalized load of 10 MR for each of the squats [33].

The load was equalized after 30 min for the next type of squat to be performed. The preparation phase for the test was performed as the previous step, including the position of the feet and barbell. The tests and collection of electromyographic data were carried out in the same shift, according to the availability of the participants, to avoid possible biases in their performance relative to variations in strength resulting from the circadian cycle.

The placement and location of electrodes followed the standardization proposed by SENIAM (Surface ElectroMyoGraphy for the Non-Invasive Assessment of Muscles) [41], available at www.seniam.org. The electrode placement sites underwent the following procedures: shaving, skin abrasion, and cleaning of the area with 70% isopropyl alcohol. The evaluated muscles and their respective positions were: (1) RF muscle: the electrodes were positioned at 50% of the anterior superior iliac spine line to the superior part of the patella, in the direction of the anterior superior iliac spine line to the superior part of the patella; (2) VL muscle: the electrodes were positioned at 2/3 of the anterior superior iliac spine line to the lateral border of the patella; (3) GM muscle: the electrodes were positioned at 50% of the line between the sacral vertebrae and the greater trochanter of the femur; and (4) long head of the BF muscle: the electrodes were positioned at 50% of the line between the ischial tuberosity and the lateral epicondyle of the tibia.

An A/D SAS1000 V8 converter (EMGSystem do Brasil—São José dos Campos, SP, Brazil) connected to a computer was used to acquire electromyographic signals. A four-channel electromyograph composed of a bipolar differential amplifier, with rejection mode (CMRR) > 85 dB, input impedance = 10 MΩ, and noise rate < 1 µV RMS according to ISEK36 was used to collect and process the myoelectric signals. The data acquisition frequency was 2000 Hz. After the acquisition, the data were stored and treated with a 20–500-Hz bandpass filter and then converted into root mean square (RMS) through a routine in the software DasyLab v. 11 (National Instruments, Dublin, Ireland). The EMG values for each muscle and exercises were normalized as a percentage of the highest EMG value produced by that muscle [42].

The electromyographic values used for analysis consisted of the third to eighth repetition to avoid signal collection both in the initial and final phases of the movement [10] since muscle fatigue alters the recruitment strategy of the quadriceps muscle group [43]. An electrogoniometer was attached to the opposite limb to measure the angle of the knee joint. This equipment is synchronized with the EMG data, allowing the identification of the ascending and descending phases of the movement.

### 4.3. Statistical Analysis

Descriptive statistics (mean ± standard deviation) were used to identify the activation of each muscle (% RMS) in the different phases (concentric and eccentric) and squat ranges (90° and 140°). The phases and angles of the movement for each muscle were considered fixed factors and the repeated measures two-way ANOVA was used to compare %RMS. Bonferroni posthoc test was used to identify differences when the interaction (F) was significant. The effect size was calculated by the partial eta squared (η^2^), adopting three classifications: small (0.0–0.25), medium (0.26–0.40), or large (>0.40). The data were analyzed using the software SPSS 18.0 (SPSS Inc., Chicago, IL, USA), with a 5% significance level.

## 5. Conclusions

The activation of the biarticular muscles RF and BF was not influenced by the squat range during the concentric action, but it was influenced during the eccentric action, with more activation at 90°. In contrast, the monoarticular muscles were influenced by the squat range in both concentric and eccentric actions. GM presented higher activation at 90° in both phases of movement, while the concentric action generated higher activation at 140° and the eccentric action generated higher activation at 90° for VL. Regarding the phases of movement, the concentric generated the highest muscle activation in all evaluated muscles. Future studies should focus on the intervening factors of movement, such as joint limitations, low back pain, and other specificities prevalent in women, in addition to checking other instrumental conditions for the exercise, such as the type of squat, the load shifted, and the machine used.

## Figures and Tables

**Table 1:** Muscular activation and effect size in squat performed by women at 90° and 140° of range of motion (*n* = 35).

	Concentric Action				Eccentric Action				Interaction			
	90°	140°	*p*	∆	90°	140°	*p*	∆	F_(1,34)_	*p*	η^2^	E.S.
Rectus femoris (RF)	52.8 ± 7.1	55.6 ± 6.7	0.097	2.8	41.3 ± 6.8	36.2 ± 8.1	0.001	5.1	37.9	0.001	0.527	Larger
Vastus lateralis (VL)	55.5 ± 8.5	59.4 ± 6.2	0.005	3.9	41.3 ± 6.9	37.6 ± 7.6	0.006	3.7	32.0	0.001	0.485	Larger
Gluteus maximus (GM)	55.8 ± 7.6	50.4 ± 7.1	0.020	5.4	23.2 ± 9.3	19.1 ± 6.1	0.022	4.1	0.70	0.409	0.020	Small
Biceps femoris (BF)	58.4 ± 12.0	56.9 ± 12.8	0.055	1.5	34.6 ± 12.1	30.6 ± 10.2	0.020	4.0	61.8	0.902	0.026	Medium

E.S.: effect size, *p*: significance level to ANOVA two way, ∆: difference of %RMS between 90° and 140° of range of motion in percentage points.

## Data Availability

The data presented in this study are available on request from the corresponding author.
